# Bivalirudin as rescue therapy for suspected heparin resistance and recurrent intra-procedural stent thrombosis during complex percutaneous coronary intervention: a case report

**DOI:** 10.3389/fcvm.2026.1864489

**Published:** 2026-07-09

**Authors:** Xiang Wang, Miao Dai

**Affiliations:** 1 Department of Cardiology and Jiujiang City Key Laboratory of Cell Therapy, Jiujiang NO.1 People’s Hospital, Jiujiang, Jiangxi, China; 2 Department of Geriatrics, Jiujiang NO.1 People’s Hospital, Jiujiang, Jiangxi, China; 3 Chronic Disease Management Center, Jiujiang NO.1 People’s Hospital, Jiujiang, Jiangxi, China

**Keywords:** anticoagulation, bivalirudin, heparin resistance, percutaneous coronary intervention, thrombosis

## Abstract

Heparin resistance (HR) poses a significant challenge during percutaneous coronary intervention (PCI), potentially leading to catastrophic thrombotic complications. Here, we report a case of suspected refractory HR and recurrent intra-stent thrombosis during a complex PCI for a heavily calcified lesion, despite aggressive antiplatelet therapy and glycoprotein IIb/IIIa inhibition. Subtherapeutic anticoagulation persisted despite repeated unfractionated heparin boluses [cumulative dose 11,000 U; activated clotting time (ACT) 132 s]. Switching anticoagulation to the direct thrombin inhibitor bivalirudin resulted in rapid achievement of therapeutic anticoagulation (ACT 320 s within 10 min) and marked reduction in thrombus burden. This case highlights bivalirudin as an effective rescue strategy for managing HR and preventing thrombosis progression in complex PCI settings, emphasizing the need for timely recognition and alternative anticoagulation in such high-risk scenarios.

## Introduction

Heparin resistance (HR) is an increasingly recognized clinical challenge during PCI, characterized by subtherapeutic anticoagulation despite standard or high doses of UFH (1). Proposed mechanisms include antithrombin III deficiency, elevated factor VIII, and nonspecific heparin binding (2). Failure to achieve adequate anticoagulation can lead to catastrophic procedural thrombotic complications. While bivalirudin, a direct thrombin inhibitor, is established as an alternative anticoagulant for PCI, its specific role as a rescue therapy for confirmed HR with active thrombosis is less documented. We report a case of successful bivalirudin use for refractory HR and recurrent stent thrombosis during complex PCI.

## Case description

A 67-year-old male presented with a one-week history of exertional chest tightness and palpitations, with a self-measured heart rate of 160–180 beats per minute lasting 5–10 min per episode. The patient had a one-month history of paroxysmal atrial fibrillation.

On admission, electrocardiography showed sinus rhythm without significant abnormalities. Laboratory tests indicated elevated liver enzymes (ALT 105.4 U/L, AST 111.2 U/L) and biomarkers (NT-proBNP 4,530 pg/ml, cTnT 18.09 pg/ml), with normal coagulation profiles and electrolytes (Table 1). Imaging studies-including chest computed tomography (CT), echocardiography, left atrial CT, and cardiac ultrasound-demonstrated cardiomegaly, bilateral atrial enlargement, moderate tricuspid regurgitation, and mild-to-moderate mitral regurgitation.

**Table 1:** Clinical and laboratory variables.

Blood analytes	September 10th, 2023	September 13th, 2023	September 14th, 2023	September 15th, 2023	September 16th, 2023	January 17th, 2024	Normal value
NT-proBNP	4,530	–	18,943	15,286	13,980	1,424	<125 pg/ml
Alanine aminotransferase	105.4	66	54.5	79.4	63.8	29.1	0–42 U/L
Aspartate aminotransferase	111.2	33	47.8	256.3	151.6	27.5	0–40 U/L
Creatinine	85	75	83	98	90	86	59–104 μmol/L
Creatine Kinase Isoenzyme	13	–	33	137	44	11	0–25 U/L
LDL-c	1.87	–	–	–	–	1.55	2.1–3.1 mmol/L
HDL-c	0.82	–	–	–	–	0.89	0.9–1.55 mmol/L
Total cholesterol	3.59	–	–	–	–	3.35	2.8–5.2 mmol/L
Troponin T	18.09	793	4,288	1,962	1,540	15.91	≤14 pg/ml
HbA1c%	5.7	–	–	–	–		4–6%
D-dimer	1.08	–	11.84	0.31	0.56	0.56	0–1.0 mg/L(FEU)
APTT	24.5	–	125.2	30.7	23.8	36.3	24–34 s
Fibrinogen	1.92	–	1.9	2.75	3.67	2.17	2–4g/L
Thrombin time	16.1	–	219.1	27.1	15.6	15.3	14–24 s
Prothrombin time	11.1	–	43.6	12.0	11.2	15.7	10–14 s

LDL-c, low-density lipoprotein cholesterol; HDL-c, high-density lipoproteincholesterol; APTT, activated partial thromboplastin time.

**Figure 1:**
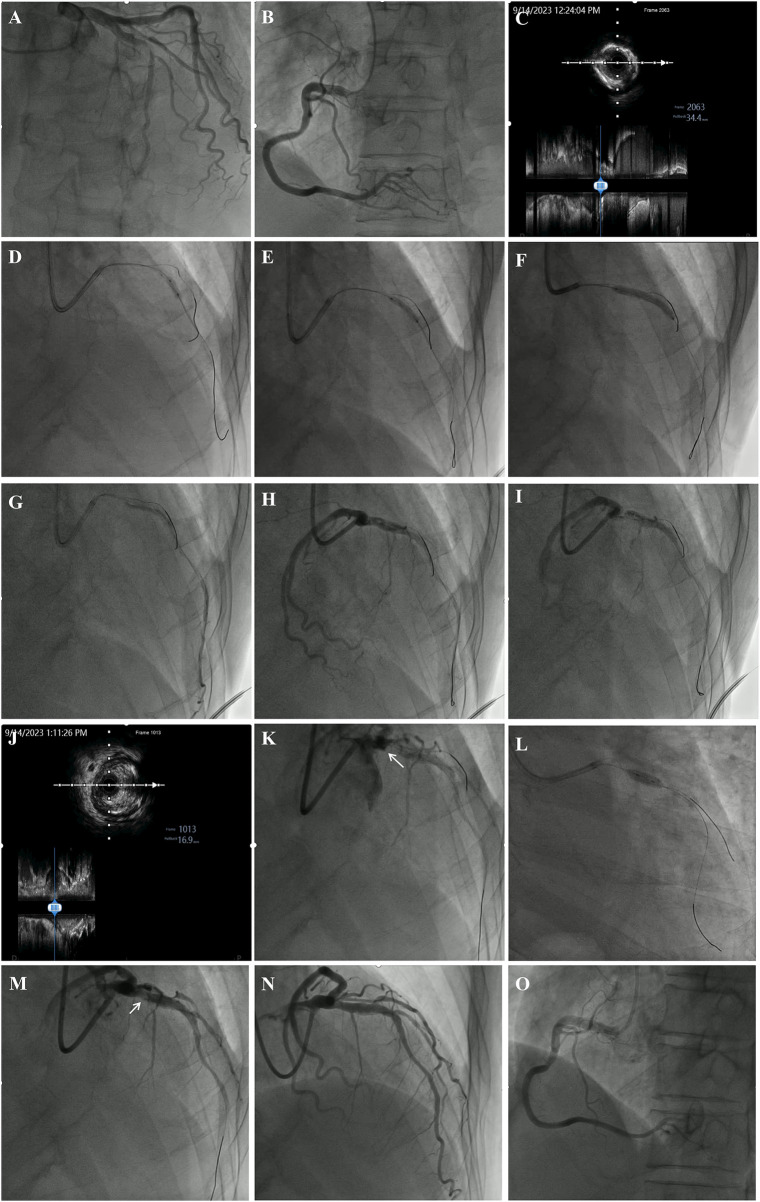
Coronary angiography and intravascular ultrasound findings during the first hospitalization and subsequent procedures. **(A)** Severe stenosis (90%) in the mid-left anterior descending artery (LADm) and ostial first diagonal branch (D1o), with 70% stenosis in the ostial second diagonal branch (D2o). Moderate stenosis (50%) in the mid-left circumflex artery (LCXm). **(B)** Moderate stenosis (50%) in the mid-right coronary artery (RCAm). **(C)** Intravascular ultrasound (IVUS) demonstrates a distal normal segment diameter of 3.7 mm and a proximal normal segment diameter of 5.0 mm in the LAD, with predominantly calcific and fibrotic lesions. **(D)** Pre-dilation of the left anterior descending (LAD) artery using a 3.0 mm cutting balloon. Contrast injection demonstrates interrupted antegrade flow (TIMI 0-1). **(E)** Failed delivery of a 3.5 × 29 mm Firebird2 stent, with stent deformation upon withdrawal due to calcific resistance. Subsequent pre-dilation with a 3.5 mm non-compliant (NC) balloon did not improve blood flow. **(F)** Successful deployment of a 3.5 × 29 mm Firebird2 stent in the LAD target lesion, followed by proximal segment post-dilation with a 4.5 mm NC balloon. Smoke injection reveals persistent absence of flow. **(G)** Microcatheter-selective angiography of the distal LAD shows a patent distal vascular bed but extensive thrombus in the mid-to-distal LAD. **(H)** Aspiration catheter retrieval of dark red thrombus, restoring TIMI 3 flow upon contrast injection. **(I)** Post-dilation with a 3.5 mm NC balloon results in reduced flow (TIMI 2). **(J)** Post-stent IVUS confirms absence of stent edge dissection but demonstrates focal underexpansion (minimal stent area 3.9 mm^2^), incomplete apposition proximally due to calcific nodules, and a high thrombus burden with intraluminal filling defects. Dense blood flow signals are also noted. **(K)** Rewiring of the diagonal branch; contrast injection indicates recurrent thrombus formation proximal to the stent (white arrow). **(L)** Delivery of a 4.5-mm non-compliant balloon to expand the proximal segment of the stent. **(M)** Angiography 10 min after bivalirudin administration shows significant thrombus reduction (white arrow). **(N)** Follow-up angiography at 4 months reveals no thrombus in the left anterior descending (LAD) artery, with a patent stent and TIMI grade 3 flow. **(O)** Follow-up angiography at 4 months demonstrates no progression of right coronary artery (RCA) disease.

Prior to the scheduled radiofrequency ablation for atrial fibrillation, coronary angiography (CAG) was performed to evaluate for possible coronary artery disease. The patient's body weight was 72 kg. After radial artery puncture, 3,000 units of unfractionated heparin (UFH) were administered. CAG revealed severe stenoses in the left anterior descending artery (LAD, 90%), first diagonal branch (D1, 90%), and second diagonal branch (D2, 70%), with moderate stenoses in the left circumflex (LCX, 50%) (Figure 1A), and right coronary artery (RCA, 50%) (Figure 1B).

Pre-percutaneous coronary intervention (PCI) antiplatelet loading included aspirin (300 mg chewed) and ticagrelor (180 mg chewed). The baseline activated clotting time (ACT) measured before any heparin administration was 118 s. ACT was monitored using a Medtronic ACT Plus Automated Coagulation Timer (USA). An additional 3,000 U of UFH was administered via the radial sheath. A 6F EBU3.5 guide catheter was positioned at the left main ostium. After wiring the LAD, D1, and D2, balloon pre-dilatation was performed (using a 2.0 mm semi-compliant balloon for the LAD and a 1.5 mm balloon for D1). Intravascular ultrasound (IVUS) confirmed the presence of a severely calcified and fibrotic lesion (distal left anterior descending artery diameter: 3.7 mm; proximal: 5.0 mm) (Figure 1C). IVUS analysis revealed extensive calcification spanning >270°, with a length of approximately 18 mm, consistent with a high calcific burden.

During PCI of the LAD using a 3.0 mm cutting balloon, the patient developed acute chest tightness, followed by abrupt flow cessation on angiography, which rapidly progressed to ventricular tachycardia/fibrillation (Figure 1D). This necessitated cardiopulmonary resuscitation (CPR) and defibrillation. A 3.5 × 29 mm Firebird2 stent encountered resistance due to the heavily calcified lesion and could not be advanced; upon withdrawal, the stent showed evidence of mechanical deformation (Figure 1E). After re-preparing the LAD with a 3.5 mm non-compliant balloon, persistent flow impairment was observed (Figure 1E). Although the stent was subsequently deployed successfully and post-dilated with a 4.5 mm balloon, coronary flow remained compromised (Figure 1F). Microcatheter angiography confirmed a patent distal vascular bed but revealed extensive thrombus in the mid-to-distal LAD (Figure 1G). Thrombectomy retrieved dark red thrombus, after which intracoronary urokinase (10 mg) and tirofiban (15 mL) were administered. Recurrent ventricular fibrillation required repeat defibrillation, after which Thrombolysis in Myocardial Infarction (TIMI) grade 3 flow was transiently achieved (Figure 1H). Subsequent balloon dilation reduced flow to TIMI 2 (Figure 1I). No edge dissection was observed (Figure 1J); however, focal stent malapposition was noted in the proximal segment due to the presence of a calcified nodule. Additionally, a thrombus shadow was visible at the proximal edge of the stent. Despite an additional bolus of tirofiban (5 mL), angiography showed recurrent proximal stent thrombus (Figure 1K). Notably, the ACT remained subtherapeutic (132 s) despite a total cumulative UFH dose of 11,000 U (including an additional 1,000 U bolus given hourly for 2 h, plus an extra 1,000 U bolus admisnistered during active thrombosis). Although antithrombin III activity and anti-Xa levels were not measured during the emergency procedure-due to the unavailability of rapid point-of-care assays in our catheterization laboratory at that time-the persistently subtherapeutic ACT despite a cumulative UFH dose of 153 U/kg supports the clinical suspicion of heparin resistance.

Anticoagulation was subsequently switched to bivalirudin (0.75 mg/kg bolus followed by a 1.75 mg/kg/h infusion), which achieved a therapeutic ACT (320 s) within 10 min. Subsequent post-dilation resulted in thrombus resolution (Figures 1L,M). The bivalirudin infusion was discontinued after 2 h due to the development of acute epigastric pain and vomit positive for occult blood, suggestive of stress-related gastric mucosal injury. No overt gastrointestinal bleeding or hemodynamic compromise occurred. Hemoglobin declined from 13.2 g/dL pre-procedure to a nadir of 12.1 g/dL at 12 h post-procedure, without need for transfusion. The patient was managed with intravenous pantoprazole (40 mg twice daily for 3 days) followed by oral pantoprazole (20 mg daily for 4 weeks), with complete symptom resolution and no further bleeding sequelae. During follow-up, the patient remained asymptomatic and compliant with his medications. A follow-up coronary angiography performed four months post-discharge, prior to ablation for paroxysmal AF, showed a patent stent without thrombus (Figures 1N,O). A detailed procedural timeline of heparin boluses, ACT values, thrombotic events, and the transition to bivalirudin is summarized in Table 2.

**Table 2:** Procedural timeline of anticoagulation, ACT values, and thrombotic events during complex PCI.

Time Point	Cumulative UFH Dose (Units)	ACT (seconds)	Event/Intervention	Antithrombotic Therapy
Baseline (pre-heparin)	0	118	Radial artery puncture; diagnostic CAG	Aspirin 300 mg + ticagrelor 180 mg (chewed)
+5 min	3,000	–	UFH bolus #1 via sheath	–
+15 min	3,000	–	Guide catheter positioning; wiring LAD, D1, D2	–
+30 min	6,000	–	Balloon pre-dilatation; IVUS	–
+45 min	6,000	–	Cutting balloon (3.0 mm) in LAD → acute chest tightness, flow cessation → VF arrest	CPR, defibrillation
+50 min	6,000	–	Stent delivery failure (“burr effect”)	–
+60 min	7,000	–	Re-preparation with 3.5 mm NC balloon → persistent flow impairment	–
+75 min	8,000	–	Stent deployed (3.5 × 29 mm) & post-dilated (4.5 mm) → flow compromised	–
+90 min	8,000	–	Microcatheter angiography: extensive thrombus in mid-distal LAD	Thrombectomy + urokinase 10 mg IC + tirofiban 15 mL IC
+95 min	8,000	–	Recurrent VF → defibrillation → transient TIMI 3 flow	–
+100 min	9,000	–	Repeat balloon → flow TIMI 2; Post-stent IVUS: MSA 3.9 mm^2^, calcium arc >270°, no edge dissection, incomplete apposition proximally with a thrombus at the proximal edge.	Additional tirofiban 5 mL IC
+105 min	10,000	–	Angiography: recurrent proximal stent thrombus	–
+110 min	11,000	132	Subtherapeutic ACT confirmed	–
+115 min	–	–	Switch to bivalirudin (0.75 mg/kg bolus + 1.75 mg/kg/h infusion)	Bivalirudin started
+125 min (10 min post-bivalirudin)	–	320	Therapeutic ACT achieved	Bivalirudin continued
+130 min	–	–	Post-dilation → thrombus resolution (Figures 1L,M)	–
+240 min (∼2 h later)	–	–	Bivalirudin infusion stopped (due to epigastric pain + occult blood)	Pantoprazole IV started
Follow-up (4 months)	–	–	Patent stent, no thrombus (Figures 1N,O)	Dual antiplatelet therapy

ACT, activated clotting time; CAG, coronary angiography; CPR, cardiopulmonary resuscitation; IC, intracoronary; IVUS, intravascular ultrasound; LAD, left anterior descending artery; NC, non-compliant; PCI, percutaneous coronary intervention; TIMI, Thrombolysis in Myocardial Infarction; UFH, unfractionated heparin; VF, ventricular fibrillation.

## Discussion

This case illustrates the critical challenge of suspected HR during complex PCI. The persistent subtherapeutic ACT despite adequate UFH dosing, in the context of a calcified, prothrombotic lesion, aligns with the diagnosis of suspected HR (3). The failure of aggressive antiplatelet and IIb/IIIa inhibitor therapy to prevent recurrent thrombosis underscored the necessity of achieving effective systemic anticoagulation.

HR during PCI is multifactorial. The most common mechanism is relative or absolute antithrombin (AT) deficiency, because UFH exerts its anticoagulant effect almost exclusively through AT binding. Acquired AT deficiency can result from preoperative UFH exposure, liver dysfunction, or consumptive coagulopathy. Alternatively, elevated levels of heparin-binding proteins such as platelet factor 4, fibrinogen, and factor VIII can sequester UFH and reduce its bioavailability. Increased heparin clearance and nonspecific binding to endothelium or macrophages may also contribute. In our patient, the persistently subtherapeutic ACT despite a cumulative UFH dose of 153 U/kg-well above the standard 70–100 U/kg for PCI-strongly supports a diagnosis of HR, although the absence of AT and anti-Xa measurements precludes definitive subclassification of the mechanism.

Bivalirudin offers several pharmacologic advantages over UFH in the setting of HR. As a bivalent direct thrombin inhibitor, it binds simultaneously to the active site and exosite-1 of thrombin, blocking both soluble and fibrin-bound thrombin without requiring AT as a cofactor (4). This AT-independent mechanism ensures predictable anticoagulation even when AT levels are reduced or when heparin-binding proteins are elevated. Bivalirudin has a linear pharmacokinetic profile and a short half-life (approximately 25 min in patients with normal renal function), allowing rapid onset of therapeutic effect and quick reversal after infusion cessation. Unlike UFH, bivalirudin does not induce thrombocytopenia (except the rare occurrence of delayed hypersensitivity reactions) and does not activate platelets. These properties make it particularly attractive for emergency rescue anticoagulation when HR is suspected (5). The 2,025 ACC/AHA/ACEP/NAEMSP/SCAI guideline recommends bivalirudin as a Class I indication in patients with STEMI undergoing PCI (6). Meta-analyses have confirmed that bivalirudin is associated with lower major bleeding and comparable ischemic events, although risk of acute stent thrombosis may be higher if the infusion is truncated prematurely (7). For HR during heart surgery, the evidence remains primarily from case series and registries, but cumulative data support bivalirudin as an effective and safe rescue strategy when UFH fails to achieve therapeutic ACT (2, 8).

In this case report, the rapid normalization of ACT after switching to bivalirudin-from 132 s to 320 s within 10 min-reflects fundamental pharmacokinetic and pharmacodynamic differences from UFH. Unlike UFH, whose activity depends on sufficient AT levels and is variably neutralized by heparin-binding proteins, bivalirudin directly binds and inhibits thrombin in a 1:1 stoichiometric ratio without any intermediary cofactor. Following an intravenous bolus (0.75 mg/kg), peak plasma concentrations are achieved almost immediately, and the ACT prolongation exhibits a linear dose-response relationship. In contrast, the effect of additional UFH boluses in a heparin-resistant patient is blunted by AT depletion or protein binding, leading to a shallow or flat dose-ACT curve. Thus, the switch to bivalirudin overcomes the limitations of AT-dependent anticoagulation, providing rapid, predictable, and profound thrombin inhibition, which was immediately reflected in the therapeutic ACT and clinical resolution of intracoronary thrombus.

It is important to note that thrombus resolution did not occur immediately after bivalirudin administration alone, but rather following a combination of continued mechanical post-dilation, prior thrombectomy, and intracoronary thrombolytic/antiplatelet therapy. Therefore, the improvement in coronary flow and thrombus burden should be interpreted as a multifactorial outcome, with bivalirudin contributing primarily by enabling rapid and sustained therapeutic anticoagulation in a setting of heparin resistance. Causality cannot be attributed solely to the switch in anticoagulant strategy.

In addition to HR, several procedural and morphological factors likely contributed to the recurrent thrombosis observed in this case. First, the target lesion was heavily calcified and fibrotic, as confirmed by IVUS. Such lesion morphology increases the risk of stent underexpansion, device delivery failure, and mechanical endothelial injury-all of which expose highly thrombogenic subendothelial structures and promote platelet activation and fibrin deposition. Second, despite successful stent deployment, transient impairment of coronary flow (TIMI 2–3 fluctuations) and angiographic evidence of slow-flow/no-reflow phenomenon were observed, likely due to microvascular obstruction from debris and platelet aggregates. Third, although we did not document overt guide catheter thrombosis, the presence of recurrent thrombus proximal to the stent raises the possibility of catheter-related or stagnant flow-related thrombosis, particularly in the setting of subtherapeutic anticoagulation. Finally, distal embolization of atherothrombotic material cannot be excluded, as thrombectomy retrieved visible thrombus and microcatheter angiography confirmed distal vessel patency following aspiration. Collectively, these factors-on a background of HR-created a highly prothrombotic milieu that was only effectively reversed after switching to bivalirudin.

A limitation of this report is the lack of antithrombin III and anti-Xa level measurement during the emergency procedure; therefore, true HR cannot be definitively distinguished from other contributors to subtherapeutic anticoagulation, including possible inadequate heparin dosing relative to body weight or a procedure-related hypercoagulable state. The total cumulative UFH dose of 11,000 U (approximately 153 U/kg) exceeded standard weight-based dosing for PCI, and the persistently low ACT despite this cumulative dose supports the clinical suspicion of HR, but we acknowledge that measurement of antithrombin III activity and anti-Xa levels would have been required for a definitive diagnosis. While we cannot confirm true heparin resistance in the absence of anti-Xa levels and antithrombin III activity, the clinical scenario-persistent subtherapeutic ACT despite escalating UFH doses exceeding 150 U/kg, with recurrent thrombosis despite adjunctive IIb/IIIa inhibition-is consistent with the operational definition of clinical heparin resistance often used in the catheterization laboratory.

This case reinforces that early recognition of heparin resistance and prompt switching to an alternative anticoagulant such as bivalirudin can help achieve therapeutic anticoagulation when heparin fails. In complex PCI with high thrombotic burden, bivalirudin may facilitate the safe application of concurrent mechanical and pharmacologic thrombus-reduction strategies, although definitive thrombus resolution in this case resulted from multiple simultaneous interventions that early recognition of HR and prompt switching to an alternative anticoagulant like bivalirudin can be crucial in preventing catastrophic complications and ensuring PCI success in complex anatomies with a high thrombotic burden.

## Data Availability

The original contributions presented in the study are included in the article/Supplementary Material, further inquiries can be directed to the corresponding author.
